# Factors Related to mHealth App Use Among Japanese Workers: Cross-Sectional Survey

**DOI:** 10.2196/54673

**Published:** 2024-10-25

**Authors:** Itsuko Ozaki, Mariko Nishijima, Eiji Shibata, Yuri Zako, Chifa Chiang

**Affiliations:** 1 Graduate School of Nursing Nagoya City University Nagoya Japan; 2 Graduate School of Medicine Ehime University Toon Japan; 3 Yokkaichi Nursing and Medical Care University Yokkaichi Japan

**Keywords:** mHealth, mobile health, mobile health apps, prevalence, health promotion, health management, Japanese worker, Japan, cross-sectional survey, disease management, app users, physical activity

## Abstract

**Background:**

Health care providers can make health guidance more effective by using mobile health technologies such as health apps. Although health care providers need to know who uses health apps, existing studies have yielded inconsistent results.

**Objective:**

The aim of the study was (1) to clarify the prevalence and patterns of health app use to improve health behaviors for preventing lifestyle-related diseases among Japanese workers and (2) to identify the associations among demographic characteristics, health behavior, and internet use and health app use by gender.

**Methods:**

Data were collected from a cross-sectional internet survey in 2023. In total, 2200 participants were included, with an even distribution of men and women in each age group aged 20 to 60 years. The participants were workers with smartphones and reported their gender, age, residence area, marital status, education, employment status, occupation, work pattern, diseases under treatment, health checkups, health guidance, health behaviors, internet use duration, and number of devices used. We asked about current and previous health app use for 1 month. A multivariate logistic regression analysis was conducted by gender.

**Results:**

Of the participants, 472 (21.5%) and 189 (8.6%) were current and previous health app users, respectively. Most current and previous health app users used features that record and track their physical activity and other health behaviors. Health app users—both men and women—were more likely to have health checkups (odds ratio [OR] 1.53, 95% CI 1.12-2.11 and OR 1.51, 95% CI 1.10-2.07, respectively), receive health guidance (OR 2.01, 95% CI 1.47-2.74 and OR 1.86, 95% CI 1.32-2.62, respectively), engage in regular physical activity (OR 2.57, 95% CI 1.91-3.47 and OR 1.94, 95% CI 1.41-2.67, respectively), use the internet for 120-179 minutes per day (OR 1.76, 95% CI 1.13-2.75 and OR 1.70, 95% CI 1.12-2.57, respectively), and were less likely to be older (50-59 years: OR 0.54, 95% CI 0.33-0.88 and OR 0.40, 95% CI 0.25-0.6, respectively, and 60-69 years: OR 0.37, 95% CI 0.22-0.62 and OR 0.47, 95% CI 0.28-0.77, respectively). According to gender, male health app users were more likely to be married (OR 1.69, 95% CI 1.23-2.33) and less likely to work in the security, agriculture, forestry, fishing, manufacturing, or transportation industries (OR 0.62, 95% CI 0.41-0.95). Female health app users were more likely to have a university education or higher (OR 1.55, 95% CI 1.061-2.26), maintain an appropriate body weight (OR 1.52, 95% CI 1.10-2.11), and use 3 or more devices (OR 2.13, 95% CI 1.41-3.23).

**Conclusions:**

Physical activity and health guidance are strong predictors of app use. Health care providers should assess the target populations’ preferences for app use based on their characteristics, support their app use, and enhance the effectiveness of health guidance.

## Introduction

Internet and communication technologies are developing rapidly worldwide. The proportion of people aged 20-59 years who use the internet in Japan is 95.2-98.4% [1]. The proportion of households with smartphones has risen from 49.5% to 88.6% in the past decade, and individuals are more likely to use a smartphone than a PC when connecting to the internet. Against this background, mobile health (mHealth) technologies have been developed and improved to support behavioral change and disease management. Thousands of health management apps (health apps) are available for smartphones, and their technical features include tracking behavior manually or semiautomatically, app communities, and app reminders [2]. In addition, these apps use behavior change techniques such as goal setting, self-monitoring, and feedback to help users improve their health habits [2]. Interventions involving health apps are increasing and are being evaluated [3-5]. A review of health app interventions reported improved physical functioning, adherence to prescribed medications, ease of symptom evaluation, and reports to care providers for people with chronic conditions [3]. In another review, most studies demonstrated significant improvements in health outcomes [4].

In Japan, one-third of men and one-fifth of women have obesity or overweight [6], leading to chronic diseases that increase medical costs. To prevent chronic diseases, promote health, and extend life expectancy, improving individuals’ lifestyles from a young age is important. Consequently, health checkups and guidance are provided to almost all segments of the population in Japan [7]. Under the Industrial Safety and Health Law, employers are required to conduct annual health checkups for full-time employees to prevent the worsening of employee health due to work. Furthermore, employers must endeavor to provide health guidance to employees who need to improve and maintain their health. Additionally, as a strategy to prevent lifestyle-related diseases, medical insurers are obligated to provide a specified health checkup to all insured persons aged between 40 and 74 years under the Act on Ensuring Medical Care for Elderly People. Based on the checkup results, specific health guidance is provided to individuals identified as having a high risk of developing lifestyle-related diseases. Thus, Japan has a unique health care system that aims to promote individuals’ efforts to improve their lifestyles based on checkup results. Health care providers, such as physicians, nurse practitioners, and dietitians, are required to provide efficient and effective health guidance. However, workers in Japan often face challenges in receiving health guidance due to time constraints. Health apps have the potential to address this issue by monitoring workers’ health-related data and facilitating message exchanges between health care providers and workers. This allows workers to receive health guidance even if they do not frequently meet with health care providers. Given the high rate of smartphone and internet use among the Japanese working population, mHealth can be used to make health guidance more efficient and effective.

Recent studies have examined the effectiveness of using an app for specific health guidance [8,9]. However, health care providers need to know which individuals use health apps to manage their health and lifestyle to effectively provide health guidance in this manner.

Several studies have examined factors associated with the use of health apps. Regarding sociodemographic factors, some studies [10-12] report that women are more likely to use or have health apps than men, while other studies [13-16] report no difference between men and women in terms of having or using health apps. Several studies [10,12,13,15,17] found that younger generations were more likely to have or use health apps, whereas other studies [11,15,16] found no differences between generations. Although Carroll et al [10] reported that individuals with greater education were more likely to have health apps, other studies [11,13-16] found no difference among educational levels. Regarding chronic conditions, Ernsting et al [13] reported that individuals with 1, 2, and 3 or more chronic conditions were more likely to use health apps than those without chronic conditions.

Conversely, Langford et al [17] reported that individuals without a self-reported history of hypertension were more likely to have health apps on their tablets or smartphones than those with a self-reported history of hypertension. Makowsky et al [14] and Robbins et al [18] found no association between health app downloads and chronic conditions. Three studies [10,13,18] that examined health apps and physical activity reported that individuals who maintained physical activity were more likely to use or have health apps than those who did not. On the contrary, Fewings et al [16] found no difference between diet-related app use and physical activity or other health behaviors such as vegetable, fruit, and fast food consumption and smoking status. However, these studies are not easily comparable because of differences in their methods and duration. Consequently, the factors associated with health app use are inconsistent across them. Moreover, few studies have examined health app use and identified its associated factors, which are expected to differ between countries because of variations in the prevalence of smartphones, health care systems, and internet literacy. mHealth technologies are developing rapidly, and attitudes toward and confidence in using health apps may change.

Against this background, we aimed (1) to clarify the prevalence and patterns of health app use to improve health behaviors and thus prevent lifestyle-related diseases among Japanese workers and (2) to identify the associations between demographic characteristics, health behavior, and internet use and health app use by gender. We focused on health apps that have functions for tracking lifestyle, body weight, and medications; searching for health information; and interacting with other users to improve health behaviors and prevent lifestyle-related diseases. Since gender differences exist in the prevalence of lifestyle-related diseases and health behaviors among Japanese citizens [6], gender may influence app use patterns. Therefore, we hypothesized that the factors related to health app use would differ between men and women. We examined which sociodemographic, health behavior, and internet use factors were associated with health app use by gender. Moreover, health care providers should consider clients’ preferences for tools that support behavior change during health guidance. For instance, understanding who prefers to use health apps for behavior change during health guidance is useful. Similarly, health care providers can more efficiently introduce health apps to clients by considering factors related to app use. In this study, we specifically focused on the current and previous use of health apps for over a period of more than a month. This is because most smartphones come with preinstalled health apps, and some apps offer a free 30-day trial. Consequently, health app users may use these apps intermittently. For example, they might temporarily stop using them once they can manage their target health behaviors or before the 30-day free trial ends.

## Methods

### Sample and Procedure

Data for this study were collected from a cross-sectional internet survey conducted by the research company Cross Marketing on February 13-14, 2023. The total number of participants was 2200. We collected data from an equal number of men and women in each age group from 20 to 60 years. Eligible participants were aged 20-69 years, were workers (including part-time work), and had a smartphone. Potential participants were sent an email asking them to complete a preliminary survey to verify their eligibility. Eligible participants were then asked to complete the main survey. The survey was terminated once the target number of participants was reached. Of the 2873 potential participants who completed the preliminary survey, 76.9% (n=2200) completed the primary survey.

All survey procedures were performed by Cross Marketing. Cross Marketing has over 5 million panelists registered across the country. They regularly collect basic information from registered panelists, which was confirmed before conducting the survey. They screened for panelists whose response times were extremely shorter than expected and excluded them. In addition, responses related to demographic characteristics were examined, and panelists with contradictory or inconsistent answers were excluded.

### Measures

#### Demographic Characteristics

We asked the participants about their gender, age, area of residence, marital status, education, employment status, occupation, and work patterns.

#### Health and Medical Services and Health Behaviors

We asked participants four questions: (1) “Do you have any diseases under treatment?” (yes or no responses), (2) “Do you have annual health checkups or physical examinations?” (yes or no responses), (3) “Have you received health guidance from a doctor, nurse, or dietician?” (3 response options ranging from 0 to ≥2), and (4) “Do you implement the following health behaviors (select all those that you implement): do not smoke, engage in regular physical activity, drink moderately or do not drink, get enough sleep each day, maintain an appropriate weight, and eat breakfast every day?” We reclassified response options into a single dichotomous outcome variable of receiving health guidance (never or more than once).

#### Internet Use

We asked participants, “Apart from using the internet for work purposes, how much time do you spend on the internet per day? (4 response options ranging from <60 to ≥180 minutes).” Participants were asked to choose all of the devices they used to access the internet on a daily basis (eg, smartphones, computers, tablets, mobile phones (excluding smartphones), internet-enabled television receivers, home game consoles, and wearable devices). We categorized the participants according to the number of devices they used: 1, 2, or 3 or more.

#### Current and Previous Use of Health Apps

The participants were asked, “Have you ever used health apps to improve health behaviors for chronic disease prevention or weight loss? (Specifically, health apps that have functions for tracking lifestyle, body weight, and medications; searching for health information; and interacting with other users to improve health behavior for weight loss or prevent lifestyle-related diseases).” The response options were “currently using health apps for over 1 month,” “have used health apps in the past for over 1 month,” and “never used health apps (including having downloaded health apps and only used them for less than 1 month).” Participants who are currently using health apps for over 1 month and those who have used health apps in the past for over 1 month were asked the following questions: “What features of the apps do you (or did you) use?” (13 response options, multiple answers), “Do you (or did you) pay to use the health apps?” (yes or no responses), “Do you (or did you) use the apps in conjunction with wearable devices (including those who do not have wearable devices)?” (yes or no responses), and “How motivated are (or were) you to use the apps?” (5 response options, multiple answers). Participants who had never used health apps (including those who downloaded health apps but only used them for less than 1 month) and those who had used health apps in the past for over 1 month but stopped using them were asked why they did not use or stopped using health apps (8 response options with multiple answers).

### Statistical Methods

Descriptive statistics were used to describe the participants’ demographic characteristics, health and medical services, health behaviors, internet use, and health app use status. We reclassified the response options into a single dichotomous outcome variable of the use of health apps. We categorized participants who are currently using health apps for over 1 month and those who have used health apps in the past for over 1 month as “users (current and previous),” and those who have never used health apps (including those who had downloaded health apps but only used them for less than 1 month) as “nonusers.” We also used the 2-tailed *t* test and chi-square test to check for differences in these factors according to gender.

We used the chi-square test to clarify the relationship between current or previous use of health apps and age, educational background, occupation, health checkups, health guidance, health behaviors, internet use duration, and number of devices used to access the internet for men and women separately. In addition, binary logistic regression analyses of data from men and women were conducted. The dependent variables were the use of health apps, and the independent variables were age, education, occupation, health checkups, health guidance, health behaviors (smoking, physical activity, drinking, sleep, appropriate weight, breakfast, and snacking), internet use duration, and number of devices used to access the internet. Odds ratios (ORs) and 95% CIs were calculated. Multivariable logistic regression analyses were conducted with all the independent variables included, and adjusted ORs and 95% CIs were calculated. Multicollinearity in multivariable logistic regression models was assessed using the variance inflation factor. Pseudo-*R*^2^ values were also calculated to determine the proportion of variance in the dependent variable that can be explained by the independent variables. Data were analyzed using SPSS Statistics (version 22; IBM Corp).

### Ethical Considerations

All the participants were asked to read the information privacy statement and informed consent form, and if they agreed to participate in the survey, they clicked an icon to begin answering the questionnaire. Completion of the internet survey was considered consent to participate in the study. Participants were not asked for any personally identifiable information. There are privacy agreements between Cross Marketing and its panelists. Participants who completed the survey were given points by Cross Marketing as a reward. Ethics approval was granted by the Research Ethics Committee of Nagoya City University, Graduate School of Nursing (ID22042-2).

## Results

### Participants’ Characteristics

The average age of the 2200 participants was 44.7 (SD 13.5; men: mean 44.9, SD 13.6 and women: mean 44.6, SD 13.3) years (Table 1). Most of the participants lived in the Southern Kanto (n=779, 35.4%), Kinki (n=355, 16.1%), and Tokai (n=299, 13.6%) regions, which included metropolitan areas. Nearly half of the participants were unmarried (n=1126, 51.2%) and had an education at the university level or higher (n=1114, 50.6%). Many participants worked full-time (n=1284, 58.4%) and had fixed hours (n=1433, 65.1%). Almost all the participants had no disease under treatment (n=1818, 82.6%). Many participants had undergone health checkups (n=1370, 62.3%) and did not receive health guidance (n=1615, 73.4%). Regarding health behaviors, many participants did not smoke (n=1329, 60.4%), while a few participants did not snack (n=238, 10.8%). One-third of the participants reported using the internet for 60-119 minutes per day, and almost all used smartphones or PCs to do so (n=1972, 89.6% and n=1429, 65%, respectively).

There were statistically significant differences in the participants’ demographic characteristics according to gender. More men than women were married, had a university education or higher, worked full-time, and engaged in flexible or discretionary work. The occupation among men was more likely to be security or agriculture, forestry, fishery, production, transportation, and construction, while among women, it was more likely to be office work. In terms of health behaviors, men were more likely to engage in regular physical activity and not snack, while women were more likely not to smoke or maintain an appropriate weight. Men were more likely to spend more time using the internet and to use more devices than women.

**Table 1:** Demographic characteristics, health-related factors, and internet use according to gender.

			All		Men		Women		*P* value	
**Age group (years), n (%)**										—^a^
	20-29	440 (20)		220 (20)		220 (20)				
	30-39	440 (20)		220 (20)		220 (20)				
	40-49	440 (20)		220 (20)		220 (20)				
	50-59	440 (20)		220 (20)		220 (20)				
	60-69	440 (20)		220 (20)		220 (20)				
Age (years), mean (SD)			44.7 (13.5)		44.9 (13.6)		44.6 (13.3)		.64^b^	
**Area of residence, n (%)**										.22^c^
	Hokkaido	110 (5)		46 (4.2)		64 (5.8)				
	Tohoku	128 (5.8)		61 (5.5)		67 (6.1)				
	Northern Kanto Koshin	111 (5)		52 (4.7)		59 (5.4)				
	Southern Kanto	779 (35.4)		410 (37.3)		369 (33.5)				
	Hokuriku	82 (3.7)		40 (3.6)		42 (3.8)				
	Tokai	299 (13.6)		146 (13.3)		153 (13.9)				
	Kinki	355 (16.1)		181 (16.5)		174 (15.8)				
	Chugoku	117 (5.3)		53 (4.8)		64 (5.8)				
	Shikoku	59 (2.7)		37 (3.4)		22 (2)				
	Kyusyu Okinawa	160 (7.3)		74 (6.7)		86 (7.8)				
**Marital status, n (%)**										.04^c^
	Unmarried	1126 (51.2)		538 (48.9)		588 (53.5)				
	Married	1074 (48.8)		562 (51.1)		512 (46.5)				
**Education, n (%)**										<.001^c^
	High school or below	610 (27.7)		295 (26.8)		315 (28.6)				
	College or vocational college	476 (21.6)		141 (12.8)		335 (30.5)				
	University or higher	1114 (50.6)		664 (60.4)		450 (40.9)				
**Employment status, n (%)**										<.001^c^
	Full-time	1284 (58.4)		767 (69.7)		517 (47)				
	Self-employed, business owner	179 (8.1)		110 (10)		69 (6.3)				
	Temporary or contract worker, part-time	737 (33.5)		223 (20.3)		514 (46.7)				
**Occupation, n (%)**										<.001^c^
	Management, research, professional	345 (15.7)		283 (25.7)		62 (5.6)				
	Medical, education, welfare	337 (15.3)		93 (8.5)		244 (22.2)				
	Office	470 (21.4)		135 (12.3)		335 (30.5)				
	Sales, marketing, service	566 (25.7)		270 (24.5)		296 (26.9)				
	Security, agriculture, forestry, fishery, production, transportation, construction	435 (19.8)		297 (27)		138 (12.5)				
	Other	47 (2.1)		22 (2)		25 (2.3)				
**Work pattern, n (%)**										<.001^c^
	Fixed hours	1433 (65.1)		706 (64.2)		727 (66.1)				
	Shifts	147 (6.7)		62 (5.6)		85 (7.7)				
	Flexible or discretionary work	401 (18.2)		241 (21.9)		160 (14.5)				
	Other	219 (10)		91 (8.3)		128 (11.6)				
**Diseases under treatment, n (%)**										.29^c^
	No	1818 (82.6)		899 (81.7)		919 (83.5)				
	Yes	382 (17.4)		201 (18.3)		181 (16.5)				
**Annual medical checkups, n (%)**										.57^c^
	Yes	1370 (62.3)		692 (62.9)		678 (61.6)				
	No	830 (37.7)		408 (37.1)		422 (38.4)				
**Health guidance, n (%)**										<.001^c^
	No	1615 (73.4)		738 (67.1)		877 (79.7)				
	More than once	585 (26.6)		362 (32.9)		223 (20.3)				
**Health behaviors, n (%)**										
	Do not smoke	1329 (60.4)		630 (57.3)		699 (63.5)		.003^c^		
	Engage in regular physical activity	796 (36.2)		451 (41)		345 (31.4)		<.001^c^		
	Drink alcohol in moderation or do not drink	1008 (45.8)		496 (45.1)		512 (46.5)		.52^c^		
	Get enough sleep each day	928 (42.2)		454 (41.3)		474 (43.1)		.41^c^		
	Maintain an appropriate weight	654 (29.7)		294 (26.7)		360 (32.7)		.002^c^		
	Eat breakfast daily	1093 (49.7)		540 (49.1)		553 (50.3)		.61^c^		
	Do not eat snacks	238 (10.8)		157 (14.3)		81 (7.4)		<.001^c^		
**Internet use duration (minutes), n (%)**										<.001^c^
	<60	528 (24)		221 (20.1)		307 (27.9)				
	60-119	712 (32.4)		376 (34.2)		336 (30.5)				
	120-179	470 (21.4)		245 (22.3)		225 (20.5)				
	≥180	490 (22.3)		258 (23.5)		232 (21.1)				
**Devices for internet use (multiple answers), n (%)**										
	Cell phone	189 (8.6)		100 (9.1)		89 (8.1)		.45^c^		
	Smartphone	1972 (89.6)		992 (90.2)		980 (89.1)		.40^c^		
	Tablet	505 (23)		281 (25.5)		224 (20.4)		.004^c^		
	PC	1429 (65)		805 (73.2)		624 (56.7)		<.001^c^		
	Wearable device	73 (3.3)		47 (4.3)		26 (2.4)		.02^c^		
	Home video game consoles with internet access	182 (8.3)		121 (11)		61 (5.5)		<.001^c^		
	Portable music player with internet access	60 (2.7)		34 (3.1)		26 (2.4)		.36^c^		
	Other	6 (0.3)		4 (0.4)		2 (0.2)		.69^c^		
**Number of devices used to access the internet, n (%)**										<.001^c^
	1	705 (32)		277 (25.2)		428 (38.9)				
	2	987 (44.9)		509 (46.3)		478 (43.5)				
	≥3	508 (23.1)		314 (28.5)		194 (17.6)				

^a^Not available.

^b^Two-tailed *t* test.

^c^Chi-square test.

### Health App Use Status

Of the participants, 472 (21.5%) were current users of health apps for at least 1 month, 189 (8.6%) had used them for at least 1 month in the past, while 1539 (70%) had never used them (this last figure includes those who downloaded health apps but only used them for less than 1 month), with no significant differences according to gender (Table 2). Almost all current and previous health app users used features to track physical activity (n=487, 73.7%) and physical data, such as weight (n=256, 38.7%), sleep status (n=138, 20.9%), and food or diet (n=137, 20.7%). Among current and previous users, 84.4% (n=558) did not pay to use the health app, and 28.6% (n=103) used the health app in conjunction with wearable devices, with significant gender differences. Most users were motivated by their own desire for health management (n=504, 76.2%). Men were more likely to be motivated by referrals during health guidance and offers from their companies or health insurers than women. The reasons for not using or stopping to use health apps were that participants were unfamiliar with or uninterested in health apps and that the apps were cumbersome.

**Table 2:** Status of health app use according to gender.

				All		Men	Women		*P* value^a^	
**Use of health apps (n=2200), n (%)**										.55
	Currently using health apps for at least 1 month		472 (21.5)		239 (21.7)		233 (21.2)			
	Used health apps previously for at least 1 month		189 (8.6)		101 (9.2)		88 (8)			
	Never used health apps		1539 (70)		760 (69.1)		779 (70.8)			
**Participants who reported current and previous use of health apps (n=661)**										
	**What features of the apps do you (or did you) use? (Multiple answers), n (%)**									
		Tracking physical activity	487 (73.7)		244 (71.8)		243 (75.7)	.29		
		Tracking food or diet	137 (20.7)		64 (18.8)		73 (22.7)	.25		
		Tracking alcohol consumption	63 (9.5)		44 (12.9)		19 (5.9)	.002		
		Tracking sleep status	138 (20.9)		82 (24.1)		56 (17.4)	.04		
		Tracking physical data (eg, weight)	256 (38.7)		124 (36.5)		132 (41.1)	.23		
		Tracking medical data (eg, blood pressure and blood glucose level)	87 (13.2)		54 (15.9)		33 (10.3)	.04		
		Tracking medication	43 (6.5)		22 (6.5)		21 (6.5)	>.99		
		Automated messages, push notifications, reminders	31 (4.7)		17 (5)		14 (4.4)	.72		
		Searching for health information	61 (9.2)		29 (8.5)		32 (10)	.59		
		Earning points and coupons	118 (17.9)		59 (17.4)		59 (18.4)	.76		
		Sharing information with other users	21 (3.2)		11 (3.2)		10 (3.1)	.55		
		Competitions and contests	22 (3.3)		14 (4.1)		8 (2.5)	.28		
		Other	3 (0.5)		0 (0)		3 (0.9)	.11		
	**Do you pay to use health apps? n (%)**									.01
		Yes	103 (15.6)		66 (19.4)		37 (11.5)			
		No	558 (84.4)		274 (80.6)		284 (88.5)			
	**Do you use the apps in conjunction with wearable devices? n (%)**									.001
		Yes	189 (28.6)		116 (34.1)		73 (22.7)			
		No	472 (71.4)		224 (65.9)		248 (77.3)			
	**What motivates you to use the app? (Multiple answers), n (%)**									
		I want to use it for self-health management	504 (76.2)		244 (71.8)		260 (81)	.01		
		It was referred to me during health guidance	89 (13.5)		66 (19.4)		23 (7.2)	<.001		
		My company or health insurer provided it	62 (9.4)		43 (12.6)		19 (5.9)	.003		
		It was recommended by a family member or friend	66 (10)		34 (10)		32 (10)	>.99		
		Other	27 (4.1)		12 (3.5)		15 (4.7)	.56		
**Participants who only reported previous use of health apps (n=1728)**										
	**Reasons why they do not use health apps or why they stopped using health apps (Multiple answers), n (%)**									
		I am unfamiliar with health apps	743 (43)		370 (48.7)		373 (47.9)	>.99		
		I am not interested in health apps	511 (29.6)		283 (37.2)		228 (29.3)	.003		
		Health apps are cumbersome to use	496 (28.7)		226 (29.7)		270 (34.7)	.03		
		Health apps are difficult to use	80 (4.6)		46 (6.1)		34 (4.4)	.17		
		I am concerned about security, such as personal information leaks	121 (7)		61 (8)		60 (7.7)	.93		
		Health apps cost a lot	116 (6.7)		57 (7.5)		56 (7.2)	.92		
		My internet connection is unstable	113 (6.5)		52 (6.8)		61 (7.8)	.44		
		Other	24 (1.4)		12 (1.6)		12 (1.5)	>.99		

^a^Chi-square test.

### Relationships Between Current and Previous Health App Use and Demographic Characteristics, Health-Related Factors, and Internet Use

The results of the chi-square test indicated significant differences in terms of marital status (*P*=.001), education (*P*<.001), occupation (*P*=.001), health checkups (*P*<.001), health guidance (*P*<.001), physical activity (*P*<.001), maintaining an appropriate weight (*P*=.01), internet use duration (*P*=.03), and number of devices (*P*<.001) in health app use among men (Multimedia Appendix 1). There were significant differences in terms of age (*P*=.003), education (*P*<.001), health checkups (*P*<.001), health guidance (*P*<.001), physical activity (*P*<.001), drinking in moderation or not drinking (*P*=.04), getting enough sleep (*P*=.01), maintaining an appropriate weight (*P*<.001), eating breakfast (*P*=0.02), internet use duration (*P*=.01), and number of devices (*P*<.001) in health app use among women (Multimedia Appendix 2).

**Figure 1:**
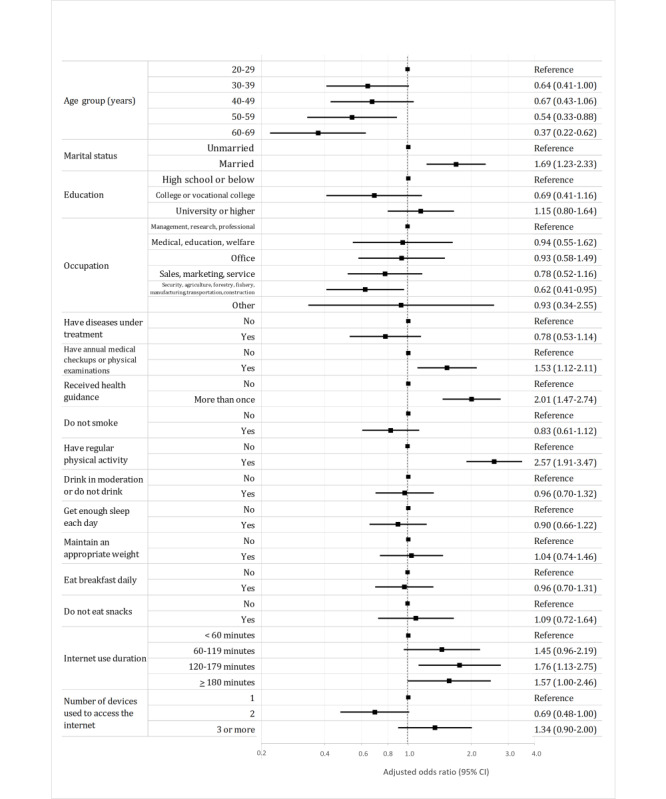
Forest plot showing adjusted odds ratios for health app use among men (n=1100).

**Figure 2:**
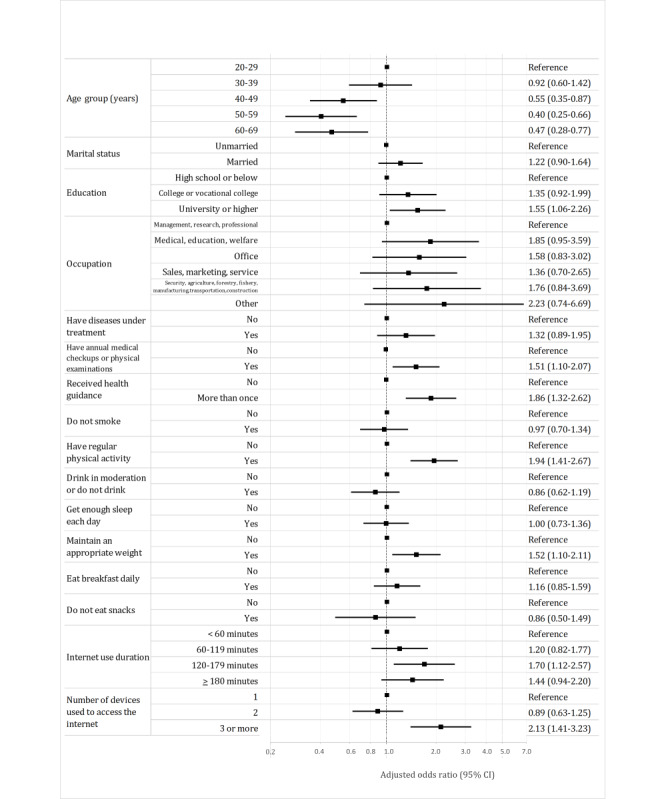
Forest plot showing adjusted odds ratios for health app use among women (n=1100).

Adjusted ORs for current and previous health app use among men and women were shown in Figures 1 and 2, respectively. The ORs for current and previous health app use were significantly higher for participants who had health checkups (men: OR 1.53, 95% CI 1.12-2.11 and women: OR 1.51, 95% CI 1.10-2.07), received health guidance (men: OR 2.01, 95% CI 1.47-2.74 and women: OR 1.86, 95% CI 1.32-2.62), engaged in physical activity (men: OR 2.57, 95% CI 1.91-3.47 and women: OR 1.94, 95% CI 1.41-2.67), and used the internet for 120-179 minutes per day (men: OR 1.76, 95% CI 1.13-2.75 and women: OR 1.70, 95% CI 1.12-2.57) and significantly lower for those aged 50-59 years (men: OR 0.54, 95% CI 0.33-0.88 and women: OR 0.40, 95% CI 0.25-0.66) and 60-69 years (men: OR 0.37, 95% CI 0.22-0.62 and women: OR 0.47, 95% CI 0.28-0.77) for both men and women. Additionally, the ORs for current and previous health app use were significantly higher for men who were married (OR 1.69, 95% CI 1.23-2.33) while being significantly lower for men who had a job in the security, agriculture, forestry, fishing, production, or transportation industries (OR 0.62, 95% CI 0.41-0.95). For women, the OR for current and previous health app use was significantly higher for those with a university education or higher (OR 1.55, 95% CI 1.061-2.26), those who maintained an appropriate weight (OR 1.52, 95% CI 1.10-2.11), and those who used 3 or more devices (OR 2.13, 95% CI 1.41-3.23). Multimedia Appendix 3 shows the results of the chi-square test and logistic regression model for the data of combined men and women. The variance inflation factor of all the independent variables in the logistic regression models ranged from 1.07 to 1.38. Therefore, multicollinearity was not present in any of the models. The *R*^2^ values were 0.186 for the model of men and 0.172 for the model of women.

## Discussion

### Principal Findings

In this study, we aimed to map and create an overview of health app use among Japanese workers and factors correlated with health app use by gender. Most current and previous health app users used features that record and track their physical activity and other health behaviors. Health app users were more likely to be younger, receive health guidance, engage in regular physical activity, and spend more time on the internet for both men and women. In terms of gender differences, male health app users were less likely to be married and less likely to work in the security, agriculture, forestry, fishing, production, or transportation industries, whereas female health app users were more likely to have a university education or higher, maintain an appropriate weight, and use 3 or more devices.

The proportions of current and previous health app users were 21.5% (n=472) and 8.6% (n=189), respectively. Ernsting et al [13] reported that the rate of health app use was 20.5% in a survey conducted in Germany in 2015, whereas Xie et al [11] reported that 38.4% of the respondents used health apps in a survey conducted in China between 2016 and 2017. In a survey conducted in Australia in 2020, Fewings et al [16] showed that 25.7% and 37.8% of the respondents were current and previous users of diet-related apps, respectively. These surveys were conducted between 2015 and 2022. Despite the increasing proportion of smartphone use among Japanese citizens [1], the prevalence of health app use among Japanese workers was low compared to the rates reported in these previous studies. However, our findings may have been influenced by the fact that we asked whether participants used health apps continuously for over a month.

The reasons for not using health apps and discontinuing their use were unfamiliarity with the apps and a lack of interest in them. Recording and tracking lifestyle and physical data, which app users in this study reported doing frequently, are useful for self-monitoring and are effective behavior change techniques [19]. Health apps have the potential to be effective tools for improving lifestyles, and their use can be increased by demonstrating their benefits and removing barriers to their use through easy-to-use interfaces.

App users in this study were likelier to have annual health checkups and receive health guidance than those who did not use the apps. A review of health checkups showed that general health checkups are associated with increased chronic disease recognition and treatment, risk factor control, preventive service uptake, and improved patient-reported outcomes [20]. Individuals who undergo a health checkup may reflect on their health status, intend to change their behavior, and consequently use health apps for behavior change. In our study, 13.5% (n=89) of the participants who used a health app reported being introduced to it during health guidance. Similarly, Hogan et al [15] showed that encouragement from health care team members to use an app was strongly associated with app use in a survey study. Individuals who trust the information provided by an app or website are more likely to use the app [16]. Encouragement from a health care provider to use a health app may increase trust in the app and promote its use. However, the features and contents of many apps lack a theoretical basis and safety [21]. Therefore, ensuring quality in the theoretical basis of the app and the safety of the information is crucial [2].

In this study, app users were more likely to engage in physical activity, which aligns with the results of previous studies [10,13,18]. In a qualitative study that explored the perspectives of young adults on apps related to behavior change, Dennison et al [22] showed that the appeal and usefulness of apps depend on whether a user is already motivated to change their lifestyle. Features such as self-monitoring tools and app reminders encourage motivated individuals to change their behavior; app use then helps cement behavior change. However, determining a causal relationship between app use and physical activity engagement based on the results of our cross-sectional survey is challenging, as app users may include individuals with greater motivation and interest in physical activity who are already engaging in physical activity [10,18].

We found no correlation between app use and diseases under treatment, similar to previous studies [14,18]. Individuals who use digital self-tracking tools are motivated to maintain their well-being rather than monitor or mediate medical problems or illnesses, and many monitor their progress in fitness or athletic training [23]. App users may include individuals willing to use the app to improve their health status and maintain their well-being by engaging in physical activity, regardless of whether they are undergoing treatment for any diseases. In this study, women using the app were also likely to maintain an appropriate weight. Weight is an important and easy-to-understand health parameter and is related to aesthetic satisfaction. Female users may intend to use the app to monitor their weight and maintain their well-being.

The app users in this study were likely to spend more time on the internet. Barriers to using health apps are that they are time-consuming and burdensome [22]. Those who spend a lot of time on the internet each day may spend some of that time using a health app and may not face these issues. Conversely, those who spend less than an hour on the internet may find it challenging to use a health app because they do not have enough time to spend on the internet or because they find it burdensome. Meanwhile, those who used the internet for more than 180 minutes did not use health apps significantly. Using the internet for a long time is an unhealthy habit, and these individuals may not be interested in using apps for disease prevention.

Female app users were more likely to use more than 3 devices to access the internet. Oshima et al [24] showed that smartphone ownership was associated with access to health care services, such as looking for health information and receiving necessary medical care. People must search for information about health apps to use suitable apps. In this study, women who used multiple devices may have had higher internet literacy, could easily access information about health apps, and found their favorite apps compared to those who used smartphones only. There was no association between app use and the number of devices among men. App users are uncomfortable with apps operating without user awareness or permission and want awareness of all features and control of all app settings [22]. Therefore, people must be skilled in using devices and technologies for apps. Men were more likely to use multiple devices than women, indicating that the former may be more skilled in using devices and technologies. Thus, the use of multiple devices does not appear to have affected the use of apps by men.

### Strengths and Limitations

One strength of this study is that it included participants from diverse backgrounds nationwide. As people may use apps intermittently, we examined both current app use for more than 1 month and previous app use lasting longer than 1 month. We clarified the status of app use and the characteristics of those currently using or who have previously used health apps to improve health behaviors. We found common factors associated with app use in men and women as well as differences. Health care providers can use this information to assess whether the target population is suited to use health apps and how to motivate them to use health apps. For instance, during health checkups and health guidance, both men and women are easily motivated to use health apps for their health management, and health care providers can support them in using health apps. As for women, health care providers need to support them by asking how long they spent on the internet and how many devices they use, and then consider whether they can use health apps. Health care providers can enhance the effectiveness of health guidance by assessing whether a target population prefers to use an app based on their characteristics and by supporting them in using the app to improve their behavior.

Nonetheless, this study has several limitations. First, due to the cross-sectional design, we could not clarify a causal relationship between app use and the mentioned factors. For example, as mentioned earlier, we could not clarify whether participants who engaged in physical activity used the app to manage it or whether app users could manage their physical activity because of using the app. Second, this study involved a web-based survey conducted through a research company. Therefore, the possibility of sample bias cannot be excluded. The proportion of smartphone users among Japanese people aged between 20 and 49 years is more than 90% [1], and the participants in this study were not a specific population compared to the general population. However, people who participate in web-based surveys may be generally interested in internet technology, such as health apps. Therefore, the proportion of current and previous app users may be higher than the actual proportions in the general population.

Meanwhile, the rate of annual health checkups in this study was lower than that of the Japanese population (62.3% vs lowest in 20s: 68.4%; and highest in 50s: 77.4%) [25]. Participants may have been less interested in health behaviors or had difficulty accessing health care services, indicating that the proportion of current and previous app users may be higher in reality. Third, as we inquired regarding previous app use, recall bias is possible. Fourth, we could not clarify various situations of app use. We defined the duration of continuous app use as using an app for more than 1 month and could only clarify current or past app use. However, there are numerous health apps, and their health behavior targets vary. We expect that health app use patterns vary, with participants changing the apps they use in a short time and using multiple apps simultaneously. Future studies are needed to develop a more detailed understanding of app use for health guidance. Finally, although we focused on health-related factors (diseases under treatment, checkups, health guidance, and health behaviors) and internet use (use duration and number of devices), other related factors may influence app use. Future research should clarify the characteristics of individuals who will use a health app when it is offered to a target population and what outcomes will be achieved by using it. In addition, interviews are needed to better understand the kinds of mHealth apps that should be designed for Japanese workers.

### Conclusions

This study clarified the status of current and previous use of health apps and related factors to prevent lifestyle-related diseases among Japanese workers. Compared to previous studies, the rate of app use among Japanese workers was low. The reasons for not using apps and discontinuing their use were unfamiliarity and a lack of interest in apps. Therefore, the number of app users can be increased by promoting effective apps for lifestyle improvement and providing easy-to-use and evidence-based apps. Health app users were more likely to be younger, receive health guidance, engage in regular physical activity, and spend more time on the internet for both men and women. Physical activity was strongly associated with app use. Although we could not clarify a causal relationship between app use and engagement in physical activity, app users may be interested and actively engage in physical activity. Health guidance was also strongly associated with app use. The introduction of an app by a health care provider may increase trust in it and promote its use. Health care providers can improve the effectiveness of health guidance by assessing whether a target population prefers to use an app based on their characteristics and by supporting them in using the app to improve their behavior.

## Appendix

Multimedia Appendix 1 Relationships between current and previous use of health apps and demographic characteristics, health-related conditions, and internet use among men.

Multimedia Appendix 2 Relationships between current and previous use of health apps and demographic characteristics, health-related conditions, and internet use among women.

Multimedia Appendix 3 Relationships between current and previous use of health apps and demographic characteristics, health-related conditions, and internet use among participants.

## Abbreviations

mHealth: mobile health
OR: odds ratio
