# Supplementary material for: Factors Related to mHealth App Use Among Japanese Workers: Cross-Sectional Survey
Source: JMIR Hum Factors. 2024 Oct 25;11:e54673. doi: 10.2196/54673 (PMC11549587; doi:10.2196/54673)
Supplement: Multimedia Appendix 1 [file humanfactors_v11i1e54673_app1.docx]

|  | Users  n = 340  n (%) | Non-users  n = 760  n (%) | P value ^a^ | Unadjusted model OR ^b^ | |
| --- | --- | --- | --- | --- | --- |
|  |  |  |  | OR (95% CI) | P value |
| **Age group** | | | | | |
| 20–29 | 73 (33.2) | 147 (66.8) | .44 | ref |  |
| 30–39 | 67 (30.5) | 153 (69.5) |  | 0.88 (0.59–1.32) | .54 |
| 40–49 | 73 (33.2) | 147 (66.8) |  | 1.00 (0.67–1.49) | 1.00 |
| 50–59 | 70 (31.8) | 150 (68.2) |  | 0.94 (0.63–1.40) | .76 |
| 60–69 | 57 (25.9) | 163 (74.1) |  | 0.70 (0.47–1.06) | .10 |
| **Marital status** | | | | | |
| Unmarried | 141 (26.2) | 397 (73.8) | .001 | ref |  |
| Married | 199 (35.4) | 363 (64.6) |  | 1.54 (1.19–2.00) | .001 |
| **Education** | | | | | |
| High school or below | 73 (24.7) | 222 (75.3) | ＜.001 | ref |  |
| College or vocational college | 31 (22.0) | 110 (78.0) |  | 0.86 (0.53–1.38) | .53 |
| University or higher | 236 (35.5) | 428 (64.5) |  | 1.68 (1.23–2.28) | .001 |
| **Occupation** | | | | | |
| Management, research, professional | 114 (40.3) | 169 (59.7) | .001 | ref |  |
| Medical, education, welfare | 31 (33.3) | 62 (66.7) |  | 0.74 (0.45–1.21) | .23 |
| Office | 45 (33.3) | 90 (66.7) |  | 0.74 (0.48–1.14) | .17 |
| Sales, marketing, service | 73 (27.0) | 197 (73.0) |  | 0.55 (0.38–0.79) | .001 |
| Security, agriculture, forestry, fishery, manufacturing, transportation, construction | 70 (23.6) | 227 (76.4) |  | 0.46 (0.32–0.65) | <.001 |
| Other | 7(31.8) | 15(68.2) |  | 0.69 (0.27–1.75) | .44 |
| **Diseases under treatment** | | | | | |
| No | 278 (30.9) | 621 (69.1) | .53 | ref |  |
| Yes | 62 (30.8) | 139 (69.2) |  | 1.00 (0.72–1.39) | .98 |
| **Annual medical checkups or physical examinations** | | | | | |
| No | 91 (22.3) | 317 (77.7) | ＜.001 | ref |  |
| Yes | 249 (36.0) | 443 (64.0) |  | 1.96 (1.48–2.59) | <.001 |
| **Health guidance** | | | | | |
| No | 190 (25.7) | 548 (74.3) | ＜.001 | ref |  |
| More than once | 150 (41.4) | 212 (58.6) |  | 2.04 (1.56–2.66) | <.001 |
| **Health behaviors (Unhealthy behavior: ref）** | | | | | |
| Do not smoke | 190 (30.2) | 440 (69.8) | .55 | 0.92 (0.71–1.19) | .53 |
| Physical activity | 195 (43.2) | 256 (56.8) | <.001 | 2.65 (2.04–3.44) | <.001 |
| Alcohol consumption | 160 (32.3) | 336 (67.7) | .39 | 1.12 (0.87–1.45) | .38 |
| Enough sleep | 148 (32.6) | 306 (67.4) | .32 | 1.14 (0.88–1.48) | .31 |
| Appropriate weight | 108 (36.7) | 186 (63.3) | .01 | 1.44 (1.08–1.90) | .01 |
| Eat breakfast daily | 176 (32.6) | 364 (67.4) | .24 | 1.17 (0.90–1.51) | .24 |
| Do not eat snacks | 54 (34.4) | 103 (65.6) | .31 | 1.20 (0.84–1.72) | .31 |
| **Internet use duration** | | | | | |
| <60 minutes | 52 (23.5) | 169 (76.5) | .03 | ref |  |
| 60–119 minutes | 118 (31.4) | 258 (68.6) |  | 1.49 (1.02–2.17) | .04 |
| 120–179 minutes | 88 (35.9) | 157 (64.1) |  | 1.82 (1.21–2.73) | .004 |
| ≥180 minutes | 82 (31.8) | 176 (68.2) |  | 1.51 (1.01–2.27) | .045 |
| **Number of devices used to access the Internet** | | | | | |
| 1 | 81 (29.2) | 196 (70.8) | ＜.001 | ref |  |
| 2 | 129 (25.3) | 380 (74.7) |  | 0.82 (0.59–1.14) | .24 |
| ≥3 | 130 (41.4) | 184 (58.6) |  | 1.71 (1.21–2.41) | .002 |

^a^ chi-square test

^b^ binary multivariate logistic regression analysis

OR: odds ratio, 95％CI: 95% confidence interval
