# Supplementary material for: Factors Related to mHealth App Use Among Japanese Workers: Cross-Sectional Survey
Source: JMIR Hum Factors. 2024 Oct 25;11:e54673. doi: 10.2196/54673 (PMC11549587; doi:10.2196/54673)
Supplement: Multimedia Appendix 2 [file humanfactors_v11i1e54673_app2.docx]

|  | Users  n = 321  n (%) | Non-users  n = 779  n (%) | P value ^a^ | Unadjusted model OR ^b^ | |
| --- | --- | --- | --- | --- | --- |
|  |  |  |  | OR (95% CI) | P value |
| **Age group** | | | | | |
| 20–29 | 77 (35.0) | 143 (65.0) | .003 | ref |  |
| 30–39 | 80 (36.4) | 140 (63.6) |  | 1.06 (0.72–1.57) | .77 |
| 40–49 | 59 (26.8) | 161 (73.2) |  | 0.68 (0.45–1.02) | .06 |
| 50–59 | 49 (22.3) | 171 (77.7) |  | 0.53 (0.35–0.81) | .003 |
| 60–69 | 56 (25.5) | 164 (74.5) |  | 0.63 (0.42–0.96) | .03 |
| **Marital status** | | | | | |
| Unmarried | 164 (27.9) | 424 (72.1) | .32 | Ref |  |
| Married | 157 (30.7) | 355 (69.3) |  | 1.14 (0.88–1.48) | .31 |
| **Education** | | | | | |
| High school or below | 66 (21.0) | 249 (79.0) | ＜.001 | Ref |  |
| College or vocational college | 94 (28.1) | 241 (71.9) |  | 1.47 (1.03-2.11) | .04 |
| University or higher | 161 (35.8) | 289 (64.2) |  | 2.10 (1.51-2.93) | <.001 |
| **Occupation** | | | | | |
| Management, research, professional | 20 (32.3) | 42 (67.7) | .34 | ref |  |
| Medical, education, welfare | 82 (33.6) | 162 (66.4) |  | 1.06 (0.59–1.93) | .84 |
| Office | 100 (29.9) | 235 (70.1) |  | 0.89 (0.50–1.60) | .71 |
| Sales, marketing, service | 74 (25.0) | 222 ( 75.0) |  | 0.70 (0.39–1.27) | .24 |
| Security, agriculture, forestry, fishery, manufacturing, transportation, construction | 37 (26.8) | 101 (73.2) |  | 0.77 (0.40–1.48) | .43 |
| Other | 8 (32.0) | 17 (68.0) |  | 0.99 (0.37–2.67) | .98 |
| **Diseases under treatment** | | | | | |
| No | 259 (28.2) | 660 (71.8) | .06 | ref |  |
| Yes | 62 (34.3) | 119 (65.7) |  | 1.33 (0.95–1.86) | .10 |
| **Annual medical checkups or physical examinations** | | | | | |
| No | 96 (22.7) | 326 (77.3) | <.001 | ref |  |
| Yes | 225 (33.2) | 453 (66.8) |  | 1.69 (1.28–2.23) | <.001 |
| **Health guidance** | | | | | |
| No | 230 (26.2) | 647 (73.8) | <.001 | ref |  |
| More than once | 91 (40.8) | 132 (59.2) |  | 1.94 (1.43–2.64) | <.001 |
| **Health behaviors (Unhealthy behavior: ref** ) | | | | | |
| Do not smoke | 209 (29.9) | 490 (70.1) | .54 | 1.10 (0.84–1.44) | .49 |
| Physical activity | 146 (42.3) | 199 (57.7) | <.001 | 2.43 (1.85–3.19) | <.001 |
| Alcohol consumption | 165 (32.2) | 347 (67.8) | .04 | 1.32 (1.02–1.71) | 0.04 |
| Enough sleep | 159 (33.5) | 315 (66.5) | .01 | 1.45 (1.11–1.88) | .01 |
| Appropriate weight | 139 (38.6) | 221 (61.4) | <.001 | 1.93 (1.47–2.53) | <.001 |
| Eat breakfast daily | 179 (32.4) | 374 (67.6) | .02 | 1.37 (1.05–1.77) | .02 |
| Do not eat snacks | 27 (33.3) | 54 (66.7) | .45 | 1.23 (0.76–2.00) | .39 |
| **Internet use duration** | | | | | |
| <60 minutes | 70 (22.8) | 237 (77.2) | .01 | ref |  |
| 60–119 minutes | 96 (28.6) | 240 (71.4) |  | 1.35 (0.95–1.93) | .10 |
| 120–179 minutes | 78 (34.7) | 147 (65.3) |  | 1.80 (1.23–2.63) | .003 |
| ≥180 minutes | 77 (33.2) | 155 (66.8) |  | 1.68 (1.15–2.46) | .01 |
| **Number of devices used to access the Internet** | | | | | |
| 1 | 107 (25.0) | 321 (75.0) | <.001 | ref |  |
| 2 | 123 (25.7) | 355 (74.3) |  | 1.04 (0.77–1.40) | .80 |
| ≥3 | 91 (46.9) | 103 (53.1) |  | 2.65 (1.86–3.79) | <.001 |

^a^ chi-square test

^b^ binary multivariate logistic regression analysis

OR: odds ratio, 95% CI: 95% confidence interval
