# Supplementary material for: Factors Related to mHealth App Use Among Japanese Workers: Cross-Sectional Survey
Source: JMIR Hum Factors. 2024 Oct 25;11:e54673. doi: 10.2196/54673 (PMC11549587; doi:10.2196/54673)
Supplement: Multimedia Appendix 3 [file humanfactors_v11i1e54673_app3.docx]

|  | Users  n =661  n (%) | Non-users  n = 1539  n (%) | P value ^a^ | Unadjusted model OR ^b^ | | Adjusted model OR ^c^ | |
| --- | --- | --- | --- | --- | --- | --- | --- |
|  |  |  |  | OR (95% CI) | P value | AOR (95% CI) | P value |
| **Age group** | | | | | | | |
| male | 340 (51.4) | 760 (49.4) | .38 | ref |  | ref |  |
| female | 321 (48.6) | 779 (50.6) |  | 0.92 (0.77–1.11) | .38 | 1.20 (0.96–1.51) | .11 |
| **Age group** | | | | | | | |
| 20–29 | 150 (22.7) | 290 (18.8) | .02 | ref |  | ref |  |
| 30–39 | 147 (22.2) | 293 (19.0) |  | 0.97 (0.73–1.28) | .83 | 0.80 (0.41–1.00) | .15 |
| 40–49 | 132(20.0) | 308 (20.0) |  | 0.83 (0.62–1.10) | .19 | 0.63 (0.46–0.86) | .004 |
| 50–59 | 119 (18.0) | 321 (20.9) |  | 0.72 (0.54–0.96) | .02 | 0.48 (0.35–0.68) | <.001 |
| 60–69 | 113 (17.1) | 327 (21.2) |  | 0.67 (0.50–0.89) | .01 | 0.43 (0.30–0.61) | <.001 |
| **Marital status** | | | | | | | |
| Unmarried | 305 (46.1) | 821 (53.3) | .002 | ref |  | ref |  |
| Married | 356 (53.9) | 718 (46.7) |  | 1.34 (1.11–1.60) | .002 | 1.49 (1.18–1.80) | .001 |
| **Education** | | | | | | | |
| High school or below | 139 (21.0) | 471 (30.6) | ＜.001 | ref |  | ref |  |
| College or vocational college | 125 (18.9) | 351 (22.8) |  | 1.21 (0.91–1.59) | .19 | 1.05 (0.78–1.42) | .74 |
| University or higher | 397 (60.1) | 717 (46.6) |  | 1.88 (1.50–2.35) | ＜.001 | 1.32 (1.02–1.70) | .04 |
| **Occupation** | | | | | | | |
| Management, research, professional | 134 (20.3) | 211 (13.7) | <.001 | ref |  | ref |  |
| Medical, education, welfare | 113 (17.1) | 224 (14.6) |  | 0.79 (0.58–1.09) | .15 | 1.09 (0.76–1.57) | .63 |
| Office | 145 (21.9) | 325 (21.1) |  | 0.70 (0.53–0.94) | .02 | 0.97 (0.69–1.35) | .83 |
| Sales, marketing, service | 147 (22.2) | 419 (27.2) |  | 0.55 (0.42–0.74) | <.001 | 0.83 (0.60–1.15) | .26 |
| Security, agriculture, forestry, fishery, manufacturing, transportation, construction | 107 (16.2) | 328 (21.3) |  | 0.51 (0.38–0.70) | <.001 | 0.81 (0.57–1.14) | .22 |
| Other | 15 (2.3) | 32 (2.1) |  | 0.74 (0.39–1.41) | .36 | 1.18 (0.58–2.37) | .65 |
| **Diseases under treatment** | | | | | | | |
| No | 537 (81.2) | 1281 (83.2) | .26 | ref |  | ref |  |
| Yes | 124 (18.8) | 258 (16.8) |  | 1.15 (0.91–1.45) | .26 | 1.00 (0.76–1.31) | .99 |
| **Annual medical checkups or physical examinations** | | | | | | | |
| No | 187(28.3) | 643(41.8) | ＜.001 | ref |  | ref |  |
| Yes | 474(71.7) | 896(58.2) |  | 1.82 (1.49–2.22) | <.001 | 1.55 (1.24–1.93) | <.001 |
| **Health guidance** | | | | | | | |
| No | 262 (39.6) | 609 (39.6) | ＜.001 | ref |  | ref |  |
| More than once | 399 (60.4) | 930 (60.4) |  | 1.99 (1.63–2.43) | <.001 | 1.94 (1.55–2.43) | <.001 |
| **Health behaviors (Unhealthy behavior: ref）** | | | | | | | |
| Do not smoke | 399 (60.4) | 930 (60.4) | .98 | 1.00 (0.83–1.20) | .98 | 0.90 (0.72–1.12) | .34 |
| Physical activity | 341 (51.6) | 455 (29.6) | <.001 | 2.54 (2.10–3.06) | <.001 | 2.21 (1.78–2.74) | <.001 |
| Alcohol consumption | 325 (49.2) | 683 (44.4) | .04 | 1.21 (1.01–1.46) | .04 | 0.92 (0.74–1.16) | .49 |
| Enough sleep | 307 (46.4) | 621 (40.4) | .01 | 1.28 (1.07–1.54) | .01 | 0.93 (0.75–1.16) | .53 |
| Appropriate weight | 247 (37.4) | 407 (26.4) | <.001 | 1.66 (1.37–2.02) | <.001 | 1.26 (1.00–1.59) | .05 |
| Eat breakfast daily | 355 (53.7) | 738 (48.0) | .01 | 1.26 (1.05–1.51) | .01 | 1.04 (0.84–1.30) | .71 |
| Do not eat snacks | 81 (12.3) | 157 (10.2) | .16 | 1.23 (0.92–1.64) | .16 | 0.99 (0.72–1.36) | .94 |
| **Internet use duration** | | | | | | | |
| <60 minutes | 122 (18.5) | 406 (26.4) | <.001 | ref |  | ref |  |
| 60–119 minutes | 214 (32.4) | 498 (32.4) |  | 1.43 (1.11–1.85) | .01 | 1.31 (1.00–1.73) | .05 |
| 120–179 minutes | 166(25.1) | 304 (19.8) |  | 1.82 (1.38–2.40) | <.001 | 1.68 (1.25–2.27) | .001 |
| ≥180 minutes | 159 (24.1) | 331 (21.5) |  | 1.60 (1.21–2.11) | .001 | 1.46 (1.08–1.97) | .02 |
| **Number of devices used to access the Internet** | | | | | | | |
| 1 | 188 (28.4) | 517 (33.6) | ＜.001 | ref |  | ref |  |
| 2 | 252 (38.1) | 735 (47.8) |  | 0.94 (0.76–1.18) | .60 | 0.80 (0.63–1.03) | .09 |
| ≥3 | 221 (33.4) | 287 (18.6) |  | 2.12 (1.66–2.70) | <.001 | 1.60 (1.21–2.12) | .001 |

^a^ chi-square test

^b^ binary multivariate logistic regression analysis

^c^ multivariate logistic regression analysis

OR: odds ratio, 95％CI: 95% confidence interval, AOR: adjusted odds ratio, ref: reference
